# Improved curve fits to summary survival data: application to economic evaluation of health technologies

**DOI:** 10.1186/1471-2288-11-139

**Published:** 2011-10-10

**Authors:** Martin W Hoyle, William Henley

**Affiliations:** 1 Peninsula College of Medicine and Dentistry, Veysey Building, Salmon Pool Lane, Exeter, EX2 4SG, UK; 2 Centre for Health and Environmental Statistics, University of Plymouth, Drake Circus, Plymouth, PL4 8AA, UK

## Abstract

**Background:**

Mean costs and quality-adjusted-life-years are central to the cost-effectiveness of health technologies. They are often calculated from time to event curves such as for overall survival and progression-free survival. Ideally, estimates should be obtained from fitting an appropriate parametric model to individual patient data. However, such data are usually not available to independent researchers. Instead, it is common to fit curves to summary Kaplan-Meier graphs, either by regression or by least squares. Here, a more accurate method of fitting survival curves to summary survival data is described.

**Methods:**

First, the underlying individual patient data are estimated from the numbers of patients at risk (or other published information) and from the Kaplan-Meier graph. The survival curve can then be fit by maximum likelihood estimation or other suitable approach applied to the estimated individual patient data. The accuracy of the proposed method was compared against that of the regression and least squares methods and the use of the actual individual patient data by simulating the survival of patients in many thousands of trials. The cost-effectiveness of sunitinib versus interferon-alpha for metastatic renal cell carcinoma, as recently calculated for NICE in the UK, is reassessed under several methods, including the proposed method.

**Results:**

Simulation shows that the proposed method gives more accurate curve fits than the traditional methods under realistic scenarios. Furthermore, the proposed method achieves similar bias and mean square error when estimating the mean survival time to that achieved by analysis of the complete underlying individual patient data. The proposed method also naturally yields estimates of the uncertainty in curve fits, which are not available using the traditional methods. The cost-effectiveness of sunitinib versus interferon-alpha is substantially altered when the proposed method is used.

**Conclusions:**

The method is recommended for cost-effectiveness analysis when only summary survival data are available. An easy-to-use Excel spreadsheet to implement the method is provided.

## Background

The estimated cost-effectiveness of health technologies (e.g. drugs, medical devices, surgical procedures) or public health interventions is often strongly influenced by the choice of survival curve. This is because the estimated expected costs and benefits (e.g. quality-adjusted life years) for each treatment are functions of the expected time patients stay in any particular health state, a summary feature of the survival distributions for the times patients stay in the health states. For example, the choice of both functional forms and parameters for curves to model progression-free survival and overall survival in the economic evaluations of drugs for renal cell carcinoma, for lenalidomide for multiple myeloma, and for sorafenib for hepatocellular carcinoma for the National Institute for Health and Clinical Excellence (NICE) in the UK [1-3] were subject to much debate. Indeed, often, seemingly minor changes in curve fits can have important impacts on cost-effectiveness, especially if considerable extrapolation is necessary.

Clearly, if individual patient data (IPD) are available, that is, the times of events or censorships for each patient, then these should be used for curve fitting. In cost-effectiveness analysis it is usual to estimate the survival curves by fitting a parametric survival model [4]. A variety of statistical distributions can be used to parameterise the model, with common choices including the Weibull, exponential and log-logistic distributions [5]. Choice of statistical distribution can be made by independently fitting the different models to the data by maximum likelihood, and selecting the distribution that achieves the best fit (e.g. the lowest Akaike's Information Criteria or Bayesian Information Criterion) [5]. Estimates of the mean survival time and other relevant parameters for the cost-effectiveness analysis can be calculated from the chosen model. The standard errors of the parameters and the covariance between parameters are recorded, and these are used to estimate the degree of uncertainty in cost-effectiveness via the probabilistic sensitivity analysis [4].

However, complete IPD are often unavailable for the purposes of economic evaluations, due to confidentiality, especially when the analyst is not employed by the sponsor of the relevant clinical trial, e.g. [2,6]. If so, and if there are only a small number of patients in a trial, it is sometimes possible to estimate the underlying IPD by reading off the times of the censorships, indicated by tick marks on the published Kaplan-Meier graph, and the times of events, indicated by stepped drops in the graph. However, this is rarely the case. Instead, summary survival data are very often used to inform cost-effectiveness models. Specifically, survival curves are often fit to Kaplan-Meier curves. Two very common methods are first, to fit by minimising the sum of squares of differences between the actual and expected survival probabilities at a range of time points (the "least squares" method), and second to regress some function of the survival probability against some other function of time, e.g. to fit a Weibull curve, regress ln(-ln(S(t)) vs. ln(t) (the "regression" method) [7]. These two methods are either applied separately to the Kaplan-Meier graphs for each treatment, or to one baseline treatment with the survival curve for the other treatment being estimated by applying the hazard ratio to the survival curve for the baseline treatment [2].

There are two important shortcomings with the traditional methods of fitting to summary survival data. First, the curve fits are influenced by all parts of the Kaplan-Meier curve equally, but the tails of Kaplan-Meier curves are often highly uncertain due to small numbers of patients at risk [8,9]. Furthermore, cost-effectiveness is driven by mean (as opposed to median) survival, which is strongly influenced by the tail of the survival curve.

Second, the traditional methods do not capture the true uncertainty in survival estimates. This is problematic because this is a key component of uncertainty in cost-effectiveness, which should be modelled in the probabilistic sensitivity analysis [4]. Clearly the true uncertainty is not captured when we fit curves to both treatments independently. For example, for the regression method, it is possible to estimate uncertainty only due to the uncertainty in estimates of the mean regression fit. The true uncertainty in effectiveness is more closely approximated when we fit a survival curve to the baseline treatment and estimate the curve for the other treatment by allowing for the uncertainty in the reported hazard ratio. However, this method does not capture the uncertainty in the baseline curve fit.

Here, we present a new method for estimating the underlying survival distribution from summary survival data. The approach is relevant for modeling the range of events typically considered in the analysis of cost-effectiveness of health technologies including overall survival, disease-free survival, progression-free survival and disease-specific outcomes such as time without epileptic seizure and time to institutionalization for dementia sufferers. The method could also be applied in other fields, such as economics, engineering and ecology, where there is a need to extract time-to-event information from published survival curves. First, we describe the method. Next, we use simulation to demonstrate that the method is likely to give a more accurate curve fit than using the least squares or regression methods. Finally, we apply the method to an economic evaluation of a cancer drug that was used to guide policy.

## Methods

### 1. Method of curve fitting

**Figure 1:**
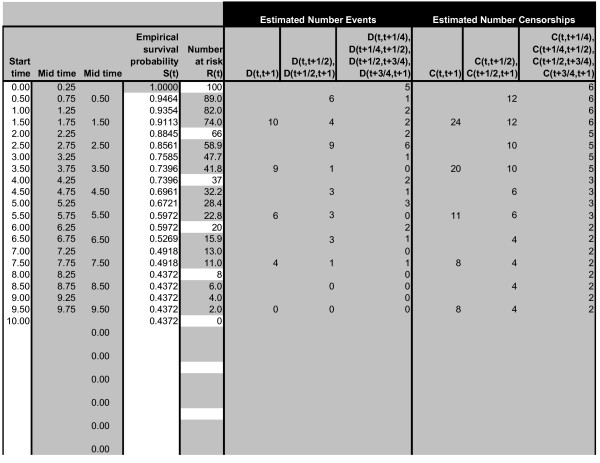
**Excel spreadsheet to estimate the numbers of patients with events and censorships per time interval**. The user needs only enter the survival probabilities from the Kaplan-Meier curve and the number of patients at risk. The R code to fit survival curves is also given in the spreadsheet.

In Step A, the method estimates the underlying IPD. This is coded in an easy-to-use Microsoft Excel spreadsheet, which is available from several sources (see Conclusions) (Figure 1). In Step B, the fitted curve is estimated by maximisation of the likelihood function for the IPD. The relevant R statistics code to estimate the survival curves is also available in the spreadsheet.

#### Step A: Estimation of underlying individual patient data

The widely cited paper by Parmar et al. [10] and the paper by Williamson et al. [11] describe a method of estimating the number of censored patients and the number of patients with events in each time interval, given the Kaplan-Meier curve. Tierney et al [12] later provided a useful spreadsheet to implement these calculations. These quantities were not used to parameterise survival curve fits (as they are in this paper), rather to estimate the hazard ratio between treatments for individual trials and then meta-analyse the hazard ratios across trials. Parmar et al. [10] and Tierney et al. [12] consider two cases: when the numbers of patients at risk at various time intervals is given, and when they are not given. In the first case, we denote the survival probabilities at each time point t from the Kaplan-Meier curve as S(t), and the number of patients at risk as R(t), in a single treatment arm in a trial with any number of treatments. R(0) is therefore the number of patients in a single treatment arm in the trial. We define the estimated number of events (e.g. deaths or clinical progression events) in each time interval [t, t+1) as D(t, t+1), and the estimated number of censorships as C(t, t+1). For simplicity, we assume that the numbers at risk are given at times t = 0, 1, 2, ...., t_max _, typically at about 5-12 time points. Then, assuming censoring is constant within each time interval [11,12];

(1a) $$S\left(t+1\right)=S\left(t\right)\left(\frac{R\left(t\right)-C\left(t,t+1\right)∕2-D\left(t,t+1\right)}{R\left(t\right)-C\left(t,t+1\right)∕2}\right)$$

R(t+1)=R(t)-C(t,t+1)-D(t,t+1)

Solving these equations [11,12];

(1b) $$D\left(t,t+1\right)=\frac{\left(R\left(t\right)+R\left(t+1\right)\right)\left(S\left(t\right)-S\left(t+1\right)\right)}{S\left(t\right)+S\left(t+1\right)}$$

$$C\left(t,t+1\right)=\frac{2\left(S\left(t+1\right)R\left(t\right)-S\left(t\right)R\left(t+1\right)\right)}{S\left(t\right)+S\left(t+1\right)}$$

A limitation of the method for estimating IPD as described by Parmar et al. [10] and Tierney et al. [12] is that the Kaplan-Meier curve can only be divided into intervals linking time points for which the numbers at risk are presented, and this may result in relatively few time points from which to estimate the survival curve. Williamson et al. [11] extended this to estimate the number of events and censorships in intervals different to those corresponding to the numbers at risk reported in the trial. The motivation was to establish time intervals common to several trials in order to estimate the pooled hazard ratio within each interval across the trials, and thus the overall pooled hazard ratio. In the next step, we use the survival probabilities at intermediate times, *S*(*t *+ 1/2), to estimate the number of events and censorships in each time interval of length 1/2. Although Williamson et al. [11] also used survival probabilities at intermediate times (corresponding to their required common time points across trials), our method differs in that we use the additional probabilities to improve estimates of the numbers of events within each interval, whereas the motivation for Williamson et al. [11] was to establish common time intervals across trials. Using the survival probabilities at intermediate times, the curve fits substantially improve, see the simulation study below. Again assuming that censoring is constant within each time interval;

(2a) $$S(t+1/2)=S(t)\left(\frac{R(t)-C(t,t+1)/4-D(t,t+1/2)}{R(t)-C(t,t+1)/4}\right)$$

$$\begin{array}{l} S(t+1)=\\ S(t+1/2)\left(\frac{R(t)-3/4C(t,t+1)-D(t,t+1/2)-D(t+1/2,t+1)}{R(t)-3/4C(t,t+1)-D(t,t+1/2)}\right) \end{array}$$

R(t+1)=R(t)−D(t,t+1/2)−D(t+1/2,t+1)−C(t,t+1)

where *D*(*t*, *t *+ 1/2) and *D*(*t *+ 1/2, *t *+ 1) are the numbers of events over the time intervals [t, t+1/2) and [t+1/2, t+1) respectively, and in general, the sum of these does not necessarily equal *D*(*t*, *t *+ 1) from Equation 1 because we use the additional information of *S*(*t *+ 1/2). We must estimate the relative sizes of the number of censorships in each interval [t, t+1/2) and [t+1/2, t+1), because otherwise, we would have three equations in four unknowns, for which there is no unique solution. Therefore, for simplicity, in Equation 2a, we assume that the rate of censoring is constant over the time interval [t, t+1). Note that, in general, *C*(*t*, *t *+ 1) in Equations 1 and 2 are not equal. The solution to these three equations in three unknowns is;

$$\begin{array}{l} D(t+1/2,t+1)=\left[S(t+1/2)-S(t+1)\right]\\ \left(\frac{S(t+1/2)R(t)+S(t+1/2)R(t+1)+2S(t)R(t+1)}{S(t+1/2)S(t)+S(t+1/2)S(t+1)+2S(t)S(t+1)}\right) \end{array}$$

$$\begin{array}{l} D(t,t+1/2)=R(t)+3R(t+1)-\left[3S(t+1)+S(t+1/2)\right]\\ \left(\frac{S(t+1/2)R(t)+S(t+1/2)R(t+1)+2S(t)R(t+1)}{S(t+1/2)S(t)+S(t+1/2)S(t+1)+2S(t)S(t+1)}\right) \end{array}$$

(2b) $$\begin{array}{l} C(t,t+1)\\ =\left[R(t)-R(t+1)-D(t,t+1/2)-D(t+1/2,t+1)\right] \end{array}$$

Also, the estimate of the number at risk at the intermediate time points is;

R(t+1/2)=R(t)−D(t,t+1/2)−C(t,t+1)/2

Next, to further improve our estimate of the number of events and censorships, we now also use the survival probabilities at intermediate times, *S*(*t *+ 1/4) and *S*(*t *+ 3/4). This allows us to estimate the number of events and censorships in each time interval of length ¼. This substantially improves the curve fits, see the simulation study below. By analogy with Equation 2b;

(3a) $$\begin{array}{l} D(t+1/4,t+1/2)=\left[S(t+1/4)-S(t+1/2)\right]\\ \left(\frac{S(t+1/4)R(t)+S(t+1/4)R(t+1/2)+2S(t)R(t+1/2)}{S(t+1/4)S(t)+S(t+1/4)S(t+1/2)+2S(t)S(t+1/2)}\right) \end{array}$$

$$\begin{array}{l} D(t+3/4,t+1)=\left[S(t+3/4)-S(t+1)\right]\\ \left(\frac{S(t+3/4)R(t+1/2)+S(t+3/4)R(t+1)+2S(t+1/2)R(t+1)}{S(t+3/4)S(t+1/2)+S(t+3/4)S(t+1)+2S(t+1/2)S(t+1)}\right) \end{array}$$

For simplicity, we also specify;

D(t,t+1/4)=D(t,t+1/2)−D(t+1/4,t+1/2)

(3b) D(t+1/2,t+3/4)=D(t+1/2,t+1)−D(t+3/4,t+1)

$$\begin{array}{l} C(t,t+1/4)=C(t+1/4,t+1/2)\\ =C(t+1/2,t+3/4)=C(t+3/4,t+1))=C(t,t+1)/4 \end{array}$$

where *C*(*t*, *t *+ 1) refers to the estimates from Equation 2b, not Equation 1. More complex expressions for *D*(*t*, *t *+ 1/4) and *D*(*t *+ 1/2, *t *+ 3/4) may be specified which are analogous to that for *D*(*t*, *t *+ 1/2) in Equation 2b, but simulation reveals no improvement in accuracy of these estimates.

The number of patients at risk over time is not always reported. In this case, the number of censorships and events in each time interval can be estimated assuming censorship only when the event has not occurred at the calendar cutoff time, and that censoring occurs at a constant rate [10,12]. A user-friendly spreadsheet for implementing this method, developed by Tierney et al [11], is given at http://www.biomedcentral.com/content/supplementary/1745-6215-8-16-S1.xls. It is recommended that the user inputs the start times of each time interval, the survival probabilities (columns C and D in worksheet "(2a)_Curve_Data") and the minimum and maximum follow up times (cells D5 and E5) and the number of patients in the trial (cell E20) into this spreadsheet. The estimated number of events and censorships in each time interval (columns G and H) are then provided. These quantities should then be input into the spreadsheet provided with this paper, as described in the spreadsheet.

#### Step B: Fitting a curve to the estimated individual patient data

In the second step, survival curves are fit to the estimated IPD, i.e. to the numbers of events and censorships in each time interval estimated in the previous step, by the method of maximum likelihood. The curves are parameterized using an appropriate probability model for survival data. Suppose we assume a two parameter distribution with parameters λ and γ (e.g. a Weibull distribution with shape parameter γ and scale parameter λ), and consider time intervals starting at t = 0, ¼, ½, ¾,..., t_max _- ¼. Then the likelihood is a product of three terms. The first term is $S{\left(t_{\max}\right)}^{R\left(t_{\max}\right)}$, because occasionally, the last reported number at risk R(t_max_), i.e. at the latest time point t_max_, which is slightly less than the maximum follow-up time, is greater than zero. Note that at the maximum follow-up time, by definition, there are no patients at risk, as all patients are censored. Therefore, if there are no patients at risk at maximum follow-up, this does not of course imply that we estimate a survival probability of zero at that time. The second term is ∏t=0,14,12,34,..,tmax−14[S(t)−S(t+14)]D(t,t+1/4), and accounts for the fact that there are *D*(*t*, *t *+ 1/4) events in the time interval [*t*, *t *+ 1/4). Here we assume the events are interval censored (i.e. we do not know precisely when the events occurred, only that they occurred in this time interval). In the simulation study below, we find that it is important to assume interval censorship for the *D*(*t*, *t *+ 1/4) events that are predicted to have occurred, because curve fits are worse when we assume that all events occur in the middle of the time intervals, i.e. at times *t *+ 1/8. The third term in the product ∏t=0,14,12,34,..,tmax−14S(t+18)C(t,t+1/4) allows for the *C*(*t*, *t *+ 1/4) censorships that are predicted to have occurred in the time interval [*t*, *t *+ 1/4). Given that we do not know the exact times of the censorships, for simplicity, we assume they occur in the middle of the time intervals, i.e. at times *t *+ 1/8. In the simulation study below, we find that this assumption is reasonable.

Combining these three terms, the likelihood is;

$$L\left(\lambda ,\gamma \right)=S{\left(t_{\max}\right)}^{R\left(t_{\max}\right)}$$

(4) ∏t=0,14,12,34,..,tmax−14[S(t)−S(t+14)]D(t,t+1/4)S(t+18)C(t,t+1/4)

The parameters $\hat{\lambda }$ and $\hat{\gamma }$ that maximize the likelihood $\hat{L}$ in Equation 4 can be estimated using standard routines in a statistical software package. Although the Weibull distribution provides a natural choice for the distribution of survival times in cost-effectiveness analysis, it is often necessary to consider alternative models, for example, to deal with situations in which the hazard function does not have a monotonic relationship with time. Other common choices include the exponential, logistic, log-normal and log-logistic distributions. One approach to selecting the best fitting curve is to chose the model which minimises Akaike's Information Criteria, given as $-2\log\hat{L}+\alpha q$[5], where *q *is the number of unknown parameters and *α *is a constant, generally taken as 2. The mean and standard deviation for each parameter, the covariance between parameters, and the Cholesky matrix, **C**, can be recorded from the output. For the cost-effectiveness model, the mean parameter values are used for the deterministic base, and for the probabilistic sensitivity analysis, the probabilistic parameters are simulated as $\hat{\Lambda }$ + **Cz**, where $\hat{\Lambda }=\left(\begin{array}{c} \hat{\lambda }\\ \hat{\gamma } \end{array}\right)$ and **z **is a vector of independent standard normal variables [4].

All calculations in Step B can be performed in R software [13] using the code provided in the online spreadsheet.

### 2. Estimating the accuracy of the method by simulation

**Figure 2:**
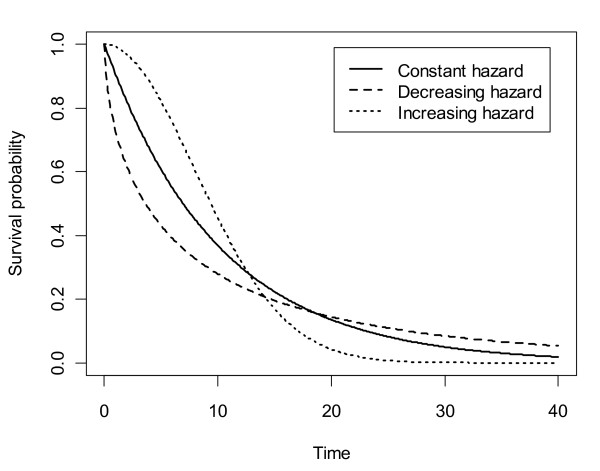
**Survivor functions for three simulated distributions**. Each distribution is parameterized by a Weibull distribution with mean time to event of 10: decreasing hazard over time (γ = 0.6, λ = 0.321), constant hazard (γ = 1, λ = 0.1), and increasing hazard (γ = 2, λ = 0.0079).

The accuracy of the proposed method was tested by simulation. Survival data were generated by simulating multiple independent trials by the Monte Carlo method in the statistics package R. Patient recruitment was modelled at a constant rate over a time period of 10 units, without loss of generality. This was also assumed to be the calendar time at maximum follow up. In this way, follow up varied from 0 to 10 time units. Patient survival was assumed to follow a Weibull distribution, *S*(*t*) = exp(-*λt^γ^*), and three shapes were independently modelled which were deemed to cover the great majority of cases experienced in practice (see Figure 2 for a plot of the survivor functions): (a): decreasing hazard over time (γ = 0.6, λ = 0.321) (e.g. patients recovering from surgery), (b) constant hazard (i.e. exponential distribution) (e.g. healthy people), (γ = 1, λ = 0.1), and (c) increasing hazard (γ = 2, λ = 0.0079) (e.g. leukaemia patients). The mean time to event was set to 10 in all three cases, corresponding to the maximum follow up time, which is typical for published survival data. The total number of patients was independently set at 100 and 500, as this covers the typical range from trials.

In addition to censoring due to patients being alive at the cut-off time, in some simulations, additional non-informative censoring was modelled at a constant hazard (e.g. to model loss to follow-up/drop out at a constant rate), with expected time of censoring equal to 5 units. Typically, the number of patients at risk are reported at about 5-12 time points. For the simulations, 6 time points were conservatively chosen, corresponding to the times 0, 2, 4, 6, 8 and 10. Survival probabilities were then recorded at time points 0, ½, 1, 1½, 2, 2½, etc, up to 10, as required from the description of the method above.

For each combination of parameter values (e.g. 100 patients, γ = 0.6, λ = 0.321, no additional censoring), 1,000 independent trials were simulated by the Monte Carlo method because this gave highly reproducible results. For each trial, the number of patients censored and the number of patients with events over each time interval of length 1/2 units were estimated from Equations 3. These were compared to the actual numbers censored and with events. The maximum likelihood estimates of the parameters of the Weibull distribution $\hat{\lambda }$ and $\hat{\gamma }$ were then estimated under the proposed method by Equation 4, and the mean time ${\left(\frac{1}{\hat{\lambda }}\right)}^{\left(\frac{1}{\hat{\gamma }}\right)}\Gamma \left(1+\frac{1}{\hat{\gamma }}\right)$ for each simulation was recorded. The mean survival time is particularly important because cost-effectiveness, as measured by the incremental cost-effectiveness ratio (ICER), is a ratio of incremental mean costs to incremental mean benefits.

Next, the performance of the proposed method was compared to that achieved by variations on the proposed method. To measure the importance of interval censoring the events, as described in the description of the method above, for some simulations, the parameters of the Weibull distribution were instead calculated assuming that all events occurred half way through the relevant interval (rather than being interval censored across the interval). Next, the importance of using the reported survival probabilities at intermediate time points was measured in the following ways. First, the number of patients censored and the number with events over each time interval of length 1 unit were calculated from Equations 2b, and the parameters of the Weibull distribution were calculated from an expression equal to Equation 4, but with half as many time intervals. Second, in a separate exercise, this procedure was repeated by calculating the number of patients censored and the number with events over each time interval of length 2 units, calculated from Equations 1b, and the parameters of the Weibull distribution calculated from an expression equal to Equation 4, but with a quarter as many time intervals. Finally, also in a separate exercise, the impact of any errors in the estimated number of patients censored and number with events over each time interval, as estimated by the proposed method in Equations 3, was measured as follows. For each simulated trial, the optimal Weibull distribution was estimated as in Equation 4, but using the actual numbers of events and censorships in each interval, instead of the estimated numbers from Equations 3.

For each combination of parameter values and for each simulated trial, the estimated mean survival time calculated by the proposed method was then compared against the estimated mean times from the following three popular alternative methods, where in each case, a Weibull distribution was chosen;

(1) Fit to the complete IPD.

(2) Fit by minimising the sums of squares of differences between the actual and estimated survival probabilities S(t) at times t = 0, ½, 1, 1½, 2, 2½, etc, up to 10.

(3) Fit by regression of log(-log(S(t))) against log(t), with S(t) again at time points 0, ½, 1, 1½, 2, 2½, etc, up to 10. The resulting slope estimates parameter γ and the intercept estimates λ.

Clearly, the first method is only possible if the full IPD are available, whereas the other two methods are commonly used in the absence of IPD. Performance of the different methods was assessed by comparing the bias and the absolute error of the mean survival times.

All the analyses above concerned estimates of the mean time. However, the uncertainty in the estimate of the mean time is a crucial determinant of the uncertainty in the cost-effectiveness of health technologies [4]. Clearly, our best estimate of the uncertainty of the mean would be calculated from the actual IPD. At the other extreme, it is impossible to estimate the uncertainty using the sums of squares and regression methods. Here, the accuracy of the estimated uncertainty in the mean using our proposed method was calculated by comparing the estimated standard error of the mean using our method against the estimated standard error of the mean using the actual IPD from simulated trials. To this effect, for each of the 1,000 simulations described in this Section, using the actual IPD, both the means of the parameters λ and γ of the Weibull distribution, and the variance-covariance matrix for these parameters were recorded. Then, for each of the 1,000 simulations, the standard error of the mean was estimated as follows. 10,000 pairs of λ and γ were randomly drawn from the means and variance-covariance matrix, and for each of these samples, the mean of the Weibull distribution was calculated. Finally, the standard deviation of these 10,000 means was calculated. This gave an estimate of the standard error of the mean for each of the 1,000 simulations. Next, this method was repeated to estimate the standard error of the mean using our proposed method for each of the 1,000 simulations. All simulations were run with γ set to 1 and for no additional censoring.

### 3. Application to cost-effectiveness of sunitinib vs. interferon-alpha for renal cell carcinoma

In this section, the proposed curve fitting method is applied to the economic evaluation of sunitinib versus interferon-alpha for renal cell carcinoma, recently performed for the National Institute for Health and Clinical Excellence (NICE) [2] in the UK. For each treatment, the following survival curves were fitted;

(a) the method originally used in the economic evaluation, by regressing ln(-ln(S(t)) against ln(t).

(b) the least squares method,

(c) the proposed method.

Next, the cost-effectiveness of sunitinib was calculated separately with these curve fits, using the original cost-effectiveness model.

## Results

### Simulation results

**Figure 3:**
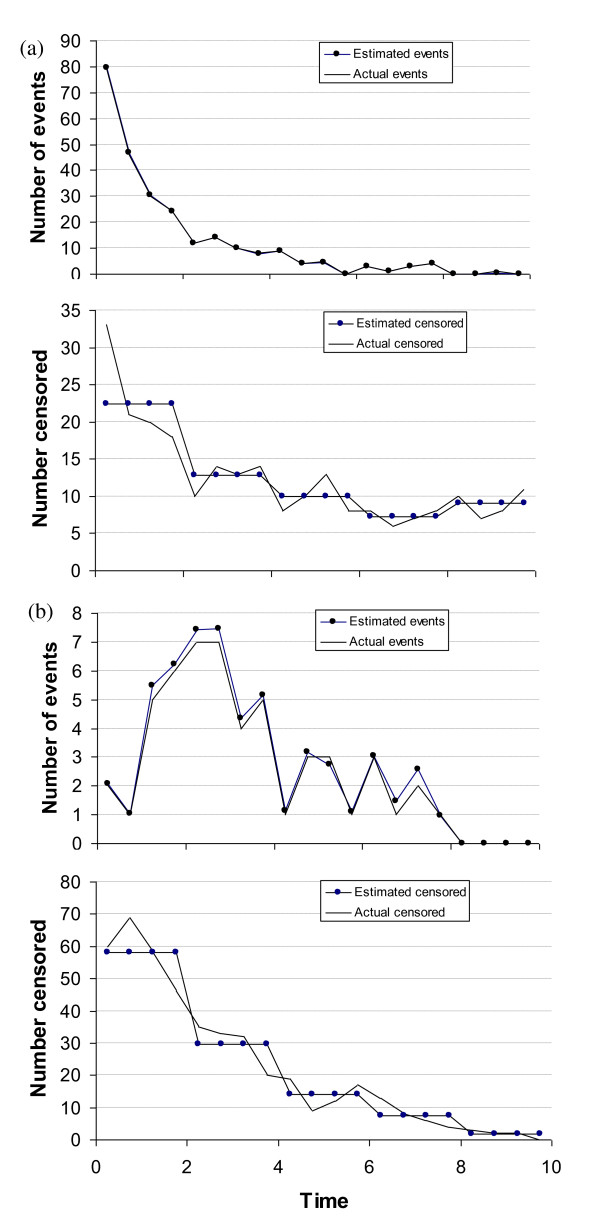
**Simulated actual versus expected numbers of events and censorships**. (a) one typical example simulated trial with decreasing hazard, 500 patients, no extra censoring, and (b) another typical example simulated trial with increasing hazard, 500 patients, with extra censoring. In (a) the curves for numbers of events are almost concurrent.

First, the proposed method accurately predicts the numbers of events and censorships in each time interval. The method is particularly accurate when there is no extra censoring (additional non-informative censoring was modelled at a constant hazard) and when the hazard decreases over time (Figure 3a), and least accurate when there is extra censoring and when the hazard increases over time (Figure 3b): the typical overestimation of the total number of events and censorships is 0% and 0% respectively for decreasing hazard, no extra censorship; 3% and -2% for decreasing hazard, with extra censorship; 1% and -0.5% for constant hazard, no extra censorship; 6% and -2% for constant hazard, with extra censorship; 2% and -0.5% for increasing hazard, no extra censorship; 7% and -0.5% for increasing hazard, with extra censorship. We believe that the accuracy of the estimated numbers of events and censorships increases with the total number of events: for the scenario in Figure 3a, there are typically approximately 265 events, and for the scenario in Figure 3b, typically 45 events. Later in this section, it is shown that any slight errors in the estimated number of events and censorships have very little impact on the accuracy of the curve fits.

**Figure 4:**
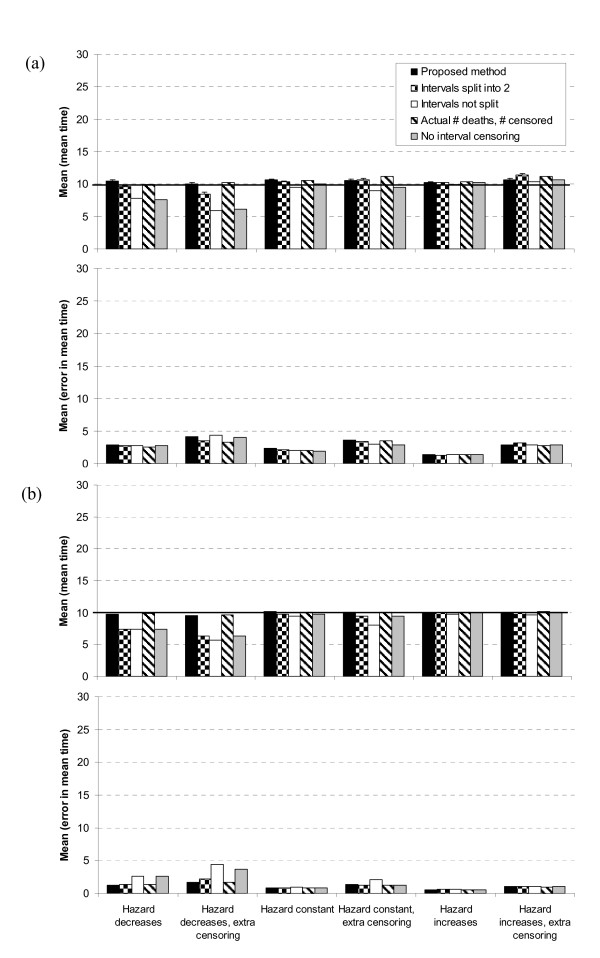
**Simulation results for variations on proposed method**. The mean, over 1,000 simulations, of the mean time and the error in the mean time for trials with (a) 100 and (b) 500 patients for the proposed method and variations on the proposed method. The population mean time is 10, as indicated by the horizontal lines. 1,000 simulations are sufficiently large that the 95% error bars (not shown) are virtually indistinguishable from the mean/median values in Figures 4, 5 and 6.

Second, we consider the performance of the proposed method in isolation (black bars in Figure 4). There is virtually no bias in estimates of the mean time (the difference between the mean over all simulations of the mean times and the population mean of 10) assuming trials of 100 and 500 patients. This is consistent with the finding that the method accurately predicts the total number of events and censorships. Estimates of the mean error in the mean time (mean absolute error) are displayed because this indicates the approximate expected error in resulting estimates of cost-effectiveness due to uncertainty in the survival distribution. As expected, the mean error is greater with additional censoring and with 100 (Figure 4a) compared to 500 patients (Figure 4b).

Third, we consider the accuracy of the proposed method compared to variations on the method. The method improves markedly when the time interval is split into more and more subsections. The bias in estimates of the mean time with the proposed method (splitting intervals into 4 subsections) is less than the bias when we split each interval in to two subsections, and this bias is itself less than the bias when we do not split the intervals (Figure 4). This is particularly so when the hazard decreases over time, and with additional censoring. The mean error in mean times is similar for the three methods for trials of 100 patients (Figure 4a), whereas it is least for the proposed method for trials of 500 patients (Figure 4b).

The results using the actual simulated numbers of events and censorships per time interval are similar to the results using the proposed method (using the estimated numbers from Equations 3) (Figure 4). This is no surprise because the proposed method accurately predicts the numbers of events and censorships in each time interval.

It is clearly preferable to interval censor the times of events, rather than assume they occur in the middle of each interval (Figure 4). The bias assuming events occur in the middle of each interval is substantial when the hazard decreases over time, particularly with additional censoring. The mean error in mean times is similar for both methods for trials of 100 patients, but is lower for the proposed method with 500 patients.

**Figure 5:**
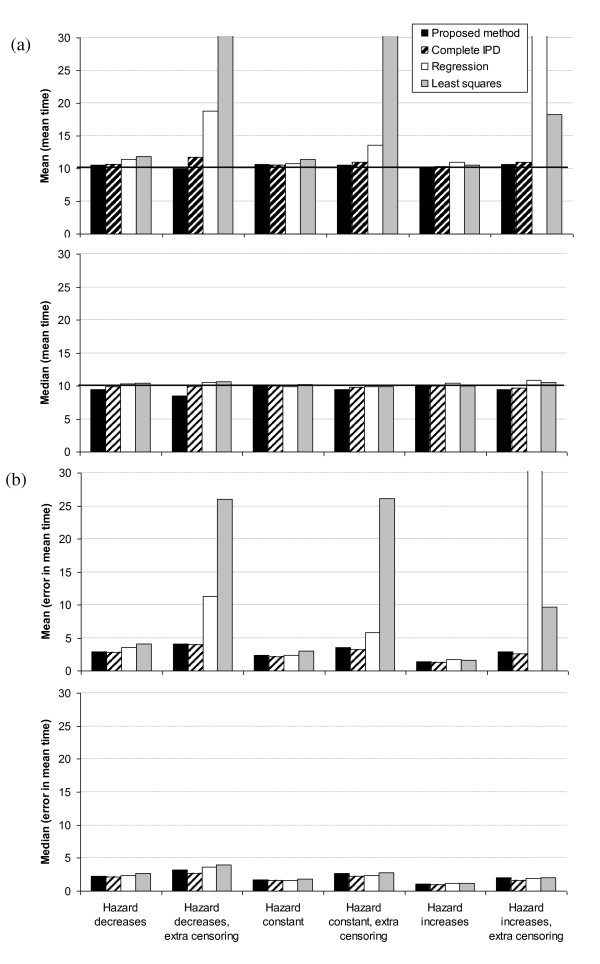
**Simulation results for proposed method vs. traditional methods: 100 patients per trial**. For 1,000 simulated trials, (a) mean(mean time) and median(mean time) and (b) mean(error in mean time) and median(error in mean time) for trials with 100 patients for the proposed method and for other established methods. The population mean time is 10, as indicated by the horizontal lines. Bars occasionally extend above 30.

**Figure 6:**
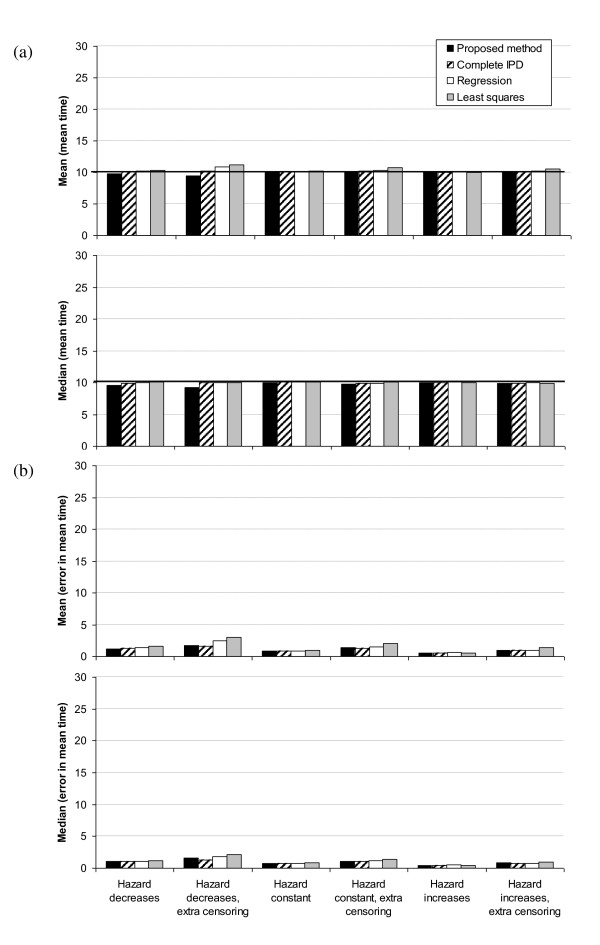
**Simulation results for proposed method vs. traditional methods: 500 patients per trial**. As Figure 5, but for trials of 500 patients.

**Figure 7:**
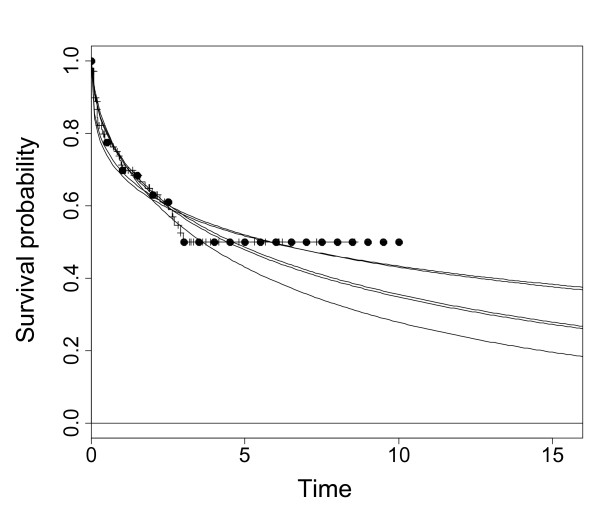
**Example simulated trial**. An example of a simulated trial (extra censoring, decreasing hazard) when the regression and least squares methods greatly overestimate the mean time. The Kaplan-Meier curve is the stepped function with tick marks indicating censorships. The time intervals are 0.5 units, indicated by the filled dots. The least squares and regression methods fit to these filled dots, and their fitted curves are the top two continuous lines, which are almost co-incident. The curves fit by maximum likelihood applied to the IPD and by the proposed method and are shown by the other pair of continuous lines, also almost co-incident. The underlying population Weibull distribution is shown by the lowest continuous curve.

Fourth, the accuracy of the proposed method is compared to three alternative established methods for trials of 100 patients (Figure 5) and 500 patients (Figure 6). First, considering 100 patients, it is immediately obvious that the mean of the estimates of the mean time for the regression and least methods are vastly over-estimated when we allow for extra censoring, although the median of the mean estimates are very accurate (Figure 5). This is because both methods occasionally greatly over-estimate the mean time when there are relatively few events at long follow up times, and because the methods give equal weight to the Kaplan-Meier curve at long and short follow up times (Figure 7). Neither the proposed method nor fitting survival curves directly to the underlying IPD suffer from this problem.

**Figure 8:**
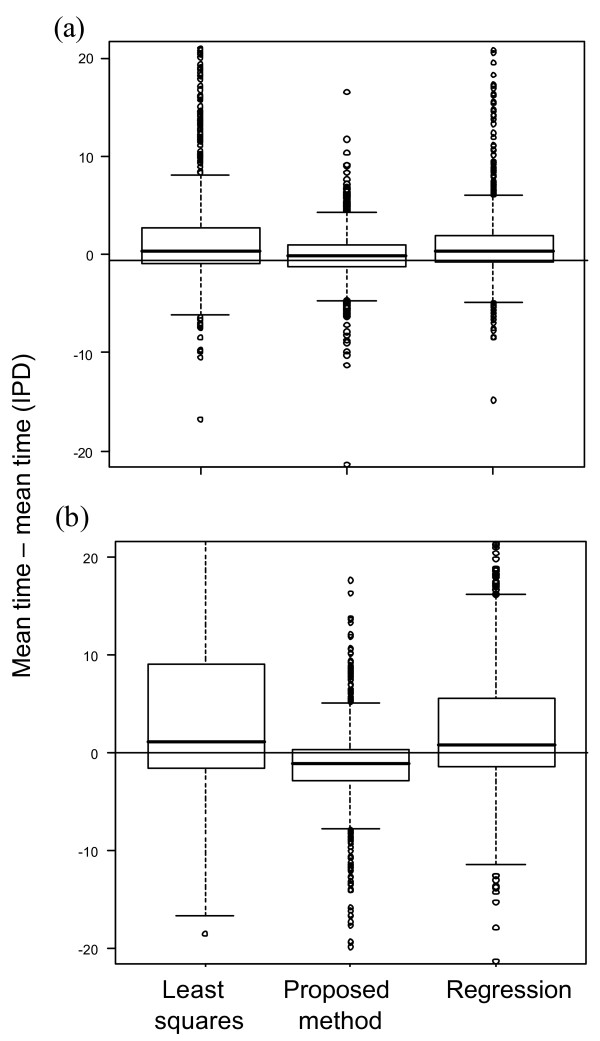
**Mean times for methods vs. mean times from IPD**. Mean times for each of the methods: least squares, proposed method, and regression, versus the mean time estimated from IPD for 1,000 simulated trials of 100 patients. Decreasing hazard is modelled (a) without and (b) with extra censoring. Some points extend outside the displayed range.

As expected, the method of fitting to the IPD performs well. However, the proposed method is about as accurate, and gives similar estimates of the mean for each simulated trial (see Figure 8, where the estimated mean for each trial based on the IPD method is closer to the estimated mean based on the proposed method than the least squares or regression methods: mean difference between proposed method and IPD = - 2% compared to corresponding mean differences of 12% and 7% for the least squares and regression methods in the absence of extra censoring). The IPD method appears to be preferable to the proposed method only when considering the median of the mean times, particularly for smaller trials, when the hazard decreases over time and with additional censoring (Figures 5).

Turning to trials of 500 patients (Figure 6), as expected, the performances of the regression and least methods are much improved. Indeed, all methods perform well. However, the proposed method very slightly underestimates the mean time when the hazard decreases and with additional censoring, and the error in the mean time for the least squares method is marginally higher in this case.

**Figure 9:**
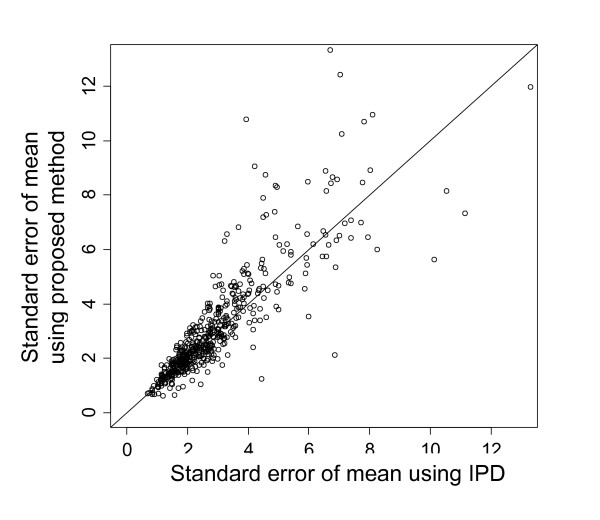
**Estimated uncertainty in mean time for proposed method vs. use of actual IPD**. 1,000 simulations were performed with the underlying distribution exponential and with no additional censoring.

Turning now to uncertainty, we find that the estimated uncertainty in the mean using our proposed method was strongly correlated with that using the actual IPD when the underlying distribution was exponential and with no additional censoring (Figure 9). This implies, at least for this combination of parameter values, that the uncertainty in the mean estimated by the proposed method is approximately as accurate as that estimated using the actual IPD.

### Application to cost-effectiveness of sunitinib vs. interferon-alpha for renal cell carcinoma

Mean progression-free survival, and hence the cost-effectiveness of sunitinib, was strongly influenced by the method of curve fitting as outlined below.

**Figure 10:**
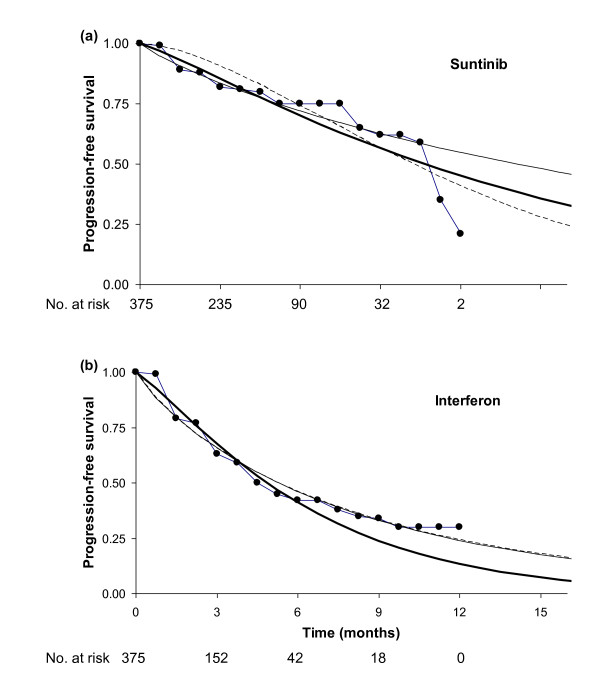
**Curve fits to progression free survival for (a) sunitinib, (b) interferon-alpha for renal cell carcinoma**. Kaplan-Meier graphs are represented by continuous stepped lines joined by dots, curves fit by the proposed method are shown by thick continuous lines, fits used in the original economic evaluation by thin continuous lines, and curves fit by least squares by dotted lines. In (b) the last two curves are almost coincident.

(a) In one of the sensitivity analyses of the original economic evaluation, a Weibull curve was fit to the Kaplan-Meier graph of progression-free survival for interferon-alpha from the Motzer et al. [14] randomised controlled trial of sunitinib versus interferon-alpha, by regressing ln(-ln(S(t)) against ln(t). Survival probabilities were taken at monthly intervals from the Kaplan-Meier graph. This yielded Weibull parameters λ = 0.16 and γ = 0.88 (Figure 10). Next, λ for sunitinib was calculated as λ for interferon-alpha multiplied by the hazard ratio of 0.42, reported in the trial [14]. γ for sunitinib was set equal to γ for interferon-alpha (Figure 10). This gave a mean progression-free survival of 8.6 months for interferon-alpha and 23.0 months for sunitinib, and an ICER of £62,000 per quality-adjusted life year (QALY) [2].

(b) Next we fit a curve to progression-free survival for interferon-alpha by least squares, using data points at 0.75-monthly intervals. This interval was chosen because the numbers of patients at risk are given at 3-monthly intervals, and for consistency with the proposed method in (c) below, we took four measurements from the Kaplan-Meier curve for each measurement of numbers at risk (see description of the method above). The interferon-alpha curve is largely unchanged, with mean progression-free survival again 8.6 months (Figure 10). Next, we independently fit a curve for sunitinib by least squares, against using data points at 0.75-monthly intervals. In this case, progression-free survival became far shorter tailed, with a mean of 11.5 months, and the ICER fell substantially, to £38,000/QALY, because patients on sunitinib are predicted to spend far less time taking the drug, as the drug is taken whilst patients are progression-free.

(c) Finally, the proposed method was applied using the survival probabilities at 0.75-monthly intervals, and the numbers at risk at 3-monthly intervals. Progression-free survival for interferon-alpha became far shorter-tailed, with a mean of 6.2 months, because this approach attaches relatively less importance to the tail of the Kaplan-Meier curve compared to the other methods, due to the small numbers of patients at risk. Progression-free survival for sunitinib, mean 13.8 months, was longer-tailed than using the least squares method, but far shorter tailed than the method used in the original evaluation. The ICER for the proposed method was £43,000/QALY. The results of the simulations suggest that this is most likely to be the most accurate estimate of the ICER. Furthermore, only this method provides an accurate estimate of the uncertainty in the curve fit, which is essential for the probabilistic sensitivity analysis.

The results of the proposed method, but neither the results of the hazard ratio method nor the least squares method, can be validated against other published data. First, the total estimated and actual numbers of progressions (as reported in Motzer et al [14] are very similar: for sunitinib, 95 versus 92 patients, and for interferon-alpha 165 versus 170 patients. Second, the reported hazard ratio of 0.42 [14], is very similar to the hazard ratio of 0.40 estimated by maximum likelihood, considering both treatments in the same statistical model (using a natural extension of the likelihood function defined in Equation 4). Whilst the number of patients taking sunitinib and interferon-alpha in the trial, at 375, are within the range of the simulation study (100 and 500), simulation to assess the accuracy of the proposed method assuming realistic patterns of censoring would further increase confidence in the accuracy of the method.

## Discussion

Parmar et al. [10] and Williamson et al. [11] provided methods of estimating the number of patients with events and the number censored in each time interval from summary survival data. These quantities were used to estimate the hazard ratios between treatments for individual trials, which were then meta-analysed across trials. The original contributions of this paper are (a) to use the estimates of the number of patients with events and the number censored in each time interval as a proxy for the IPD and to estimate the underlying survival distribution (and hence the mean survival time) with this estimated IPD and (b) to improve our estimates of the underlying IPD by using survival probabilities at additional time points. Contribution (b) was necessary because curves fits based on the unimproved estimates are frequently inaccurate. On the other hand, curves fit using the proposed method are very nearly as accurate as those fit from the actual IPD. Indeed, if it is not necessary to stratify by covariates such as age and sex, or to estimate the correlation between events when the trial reports more than one type of event per patient (e.g. cancer progression and death), in which case IPD is needed, then it is not clear that it is always worth obtaining the IPD.

Simulation demonstrates that all methods perform well in trials with many (500) patients, although the expected error in the estimate of the mean is slightly less with the proposed method and the IPD method compared to the traditional methods of least squares or regression. However, the proposed method, like the IPD method, provides much more stable estimates than the traditional methods for trials with few patients and considerable censoring. Six time points were conservatively chosen for the simulations, whereas the numbers at risk at 5-12 time points are typically reported. Clearly, the method will become more accurate with more time intervals. Therefore, if numbers at risk are reported at more than 6 time points, the curve fits using the proposed method will be more accurate than reported here.

One advantage of the proposed method is that it is not necessary to make the assumption of proportional hazards, because the curve fits for each treatment are estimated independently. This contrasts with the popular method of fitting a curve to the baseline treatment directly from the Kaplan-Meier graph and then estimating the curve for the other treatment by applying the hazard ratio to the baseline treatment. Another important advantage of the proposed method is that the true uncertainty in the survival curves is estimated for use in the probabilistic sensitivity analysis in the economic evaluation. This is not possible using the traditional methods of estimating survival curves from summary survival data. Simulation suggests that the uncertainty estimated by the proposed method is close to that estimated from the actual IPD (Figure 9). However, the uncertainty estimated by the proposed method will be slightly underestimated, because we are assuming the IPD in Step A (in the description of the method above) are estimated with complete certainty. However, given that the method estimates the IPD well, this inaccuracy is likely to be very slight.

The main disadvantage of the proposed method is that slightly more work is required to implement the method compared to the least squares or regression methods. However, the underlying IPD are estimated automatically using the Online spreadsheet, and the curves can be fit using the Online R statistics code with minimal input from the user. Given that the cost-effectiveness of health technologies is often strongly determined by the estimated survival curve, we believe that any extra effort is easily justified. Nonetheless, some analysts may be put off by using what may be an unfamiliar statistics package. The R package was chosen because it is freely available and provides functions to maximise the likelihood in the presence of interval censoring, (simulation demonstrates that interval censoring is important to improve the curve fits). Other widely used statistical packages such as Stata and SAS also provide procedures for estimating failure time models in the presence of interval censoring, and could be used to carry out Step B of the proposed method.

We now make some general recommendations. Given the consistent performance of the proposed method in the simulation study, we recommend it is used in preference to the least squares and regression methods regardless of the size of trial or level of censoring. This is for three reasons. First, the analyst need not consider whether the traditional methods are likely to be subject to the extreme bias seen in smaller trials with additional censoring. Second, even in large trials, there may be just a few patients with very long follow up, and these will strongly influence curve fits using the traditional methods, but not using the proposed method. Third, only the proposed method gives estimates of the true uncertainty in the curve fit.

We further recommend that either (a) the sponsor of the trial publishes the best fit underlying survival distribution estimated directly from the IPD, or (b) Kaplan-Meier graphs should always be accompanied by the numbers of patients at risk, ideally at as many time points as possible. Either way, the sponsor need not release the IPD, and therefore confidentiality of the data is maintained. The second recommendation is included because the proposed method works best when the numbers at risk are available (although the proposed method can be used without the numbers at risk, by first estimating the IPD from the spreadsheet of Tierney et al [12], then applying Equation 4).

Throughout, we have considered a single trial arm. However, clearly the method can easily be extended to allow for two treatments in a single trial. First, the IPD for both arms can be independently estimated from Equations 3. Then either the maximum likelihood estimates of the parameters can be calculated separately for each treatment using Equation 4, or a two-group parametric survival model can be fitted to the IPD for both treatment arms in a statistics package such as R. Under the second method, the estimated hazard ratio can be compared with the published hazard ratio as a validity check (as described in the section on the cost-effectiveness of sunitinib).

Alternatively, instead of using the published hazard ratio as a validity check, it is reasonable to ask whether it could be used to improve the accuracy of our survival estimates for the two treatment arms. Consider the following three methods;

1. Fit a survival curve to one of the two treatment arms using one of the traditional methods of fitting to summary survival data, i.e. the least squares method or the regression method, and then estimate the survival curve for the other treatment arm by applying the hazard ratio to the first arm.

2. Repeat the first method, but instead, fit a survival curve to one of the two treatment arms using our proposed method.

3. Fit independent survival curves to the two treatment arms using our proposed method. In this case, the published hazard ratio is not used.

This study has shown that Method 2 is superior to Method 1. Further, we believe that the hazard ratio which can be calculated from Method 3 is likely to be very similar to the published hazard ratio because we have shown that the proposed method accurately predicts the underlying IPD, which is used to calculate the published hazard ratio. Therefore, we believe that Method 3 is preferable to Method 2, given also that Method 2, but not Method 3, requires the proportional hazards assumption, which may or may not be realistic. Whilst the published hazard ratio provides a useful summary of the relative survival between the two treatments, cost-effectiveness is often driven not just by relative survival, but also by absolute survival in the two treatment arms (e.g. when the costs associated with a health state are very different between treatment arms). So far, we have assumed that the numbers of patients at risk at each of several follow-up times are available. If instead this data is not available, it is not clear which of Methods 2 or 3 are likely to be superior, given that we have not evaluated the accuracy of the proposed method by simulation when the numbers at risk are not available. Therefore, we encourage further research to answer this question.

We now suggest some further research. It is impossible to cover every possible combination of parameters in simulations. Those presented were chosen as they were deemed plausible in actual clinical trials: the underlying survival distribution was assumed to be Weibull (although the R code supplied can fit other functional forms) because of it flexibility in modeling both increasing and decreasing hazard functions: allowance was also made for variation in the number of the patients enrolled in a trial and the effect of additional censoring. Nonetheless, further research is required to explore the accuracy of the proposed method in other circumstances which are deemed relevant to actual trials, e.g. with alternative survival distributions and/or variations in the degree of censoring to reflect the levels experienced in actual trials. In this study, it has been assumed that each individual has a constant hazard of additional censoring during the study follow-up (in this way, additional censoring is non-informative, since the censoring time is statistically independent of the failure time). Other censoring mechanisms that allow for variation in the rate of censoring with time and incorporate informative drop-out may be more realistic in some contexts and could be explored. It has been shown that sub-dividing the time intervals and using survival probabilities at additional time points improves the accuracy of the method. Further work is also encouraged to quantify the improvement of the method if the time intervals are further sub-divided. One possible criticism of the simulation study is that we used the exact survival probabilities, rather than being forced to read the probabilities off published Kaplan-Meier curves. However, we believe that survival probabilities can usually be read with good accuracy. Furthermore, any inaccuracies apply equally to all methods assessed, with the exception of use of the actual IPD.

The proposed method accurately predicts the underlying distribution in the great majority of scenarios. However, the simulation study showed that the method gives estimates with a small degree of bias in some scenarios. For example, estimates of the mean survival time were biased when the sample size was 100 patients and the hazard was decreasing (e.g. estimated bias = 5% without additional censoring). These results reflect the known bias in the Weibull shape parameter when it is estimated by maximum likelihood estimation for smaller sample sizes or in the presence of heavy censoring [15]. The proposed method outperforms the traditional methods despite this bias: the relative efficiency of the proposed method relative to the IPD model was 1.02 compared to 0.19 and 0.34 for the least squares and regression methods respectively. Furthermore, in the presence of additional censoring, the relative efficiency of the proposed method relative to the IPD model improved to 1.52 compared to 0.0005 and 0.01 for the least squares and regression methods respectively.

Yang and Xie [15] propose an alternative estimator of the Weibull shape parameter based on a modified profile likelihood applied to the IPD. Through simulation, Yang and Xie demonstrate that this modified maximum likelihood estimate (MMLE) approach is almost unbiased and much more efficient than the regular MLE when the data are complete or Type II censored. In the case of Type I censoring, the MMLE approach also performs much better than regular MLE. Step B of the method of curve fitting proposed in this paper provides a flexible framework and could be extended to include use of MMLE or other relevant methods to reduce bias in situations where the regular MLE approach is known to perform poorly.

## Conclusions

We have presented a method for estimating the underlying survival distribution from a Kaplan-Meier graph. The number of patients at risk improves the accuracy of the survival distribution. Simulation demonstrates that the method provides more accurate estimates of mean survival compared to the least squares and regression methods under a plausible range of parameter values. Furthermore, application of the method to a model of the cost-effectiveness of a cancer drug demonstrates that the method may yield cost-effectiveness estimates that differ substantially from those using traditional methods. Therefore, we recommend the method in preference to the traditional methods when IPD are not available. We further recommend that published results of trials should provide not only Kaplan-Meier graphs but also the numbers of patients at risk, ideally at as many time points as possible, so that our method can be employed to greatest effect. An easy-to-use Microsoft Excel spreadsheet that implements the proposed method is available either directly from the authors or from the PenTAG website: http://sites.pcmd.ac.uk/pentag, under "Staff/Martin Hoyle".

## Competing interests

The authors declare that they have no competing interests.

## Authors' contributions

MH conceived of the study, performed the statistical analyses and wrote the first draft of the manuscript. WH tested the spreadsheet, modelled the effect of censoring on the results of the method, and helped with later drafts of the manuscript. Both authors read and approved the final manuscript.

## Authors' information

MH is a health economist and modeller at the Peninsula Technology Assessment Group (PenTAG), one of the Assessment Groups commissioned by NICE in the UK to assess the clinical and cost-effectiveness of health technologies. In this capacity, MH often fits survival distributions to summary survival data. The work in this manuscript was developed in response to this requirement.

## Pre-publication history

The pre-publication history for this paper can be accessed here:

http://www.biomedcentral.com/1471-2288/11/139/prepub
